# Holistically sustainable continence care: A working definition, the case of single-used absorbent hygiene products (AHPs) and the need for ecosystems thinking

**DOI:** 10.1177/09544119231188860

**Published:** 2023-09-01

**Authors:** Tiina Vaittinen, Krista Koljonen, Susanna Tella, Eveliina Asikainen, Katri Laatikainen

**Affiliations:** 1 Faculty of Management and Business (Politics Unit) and Faculty of Social Sciences (Global Health and Development), Tampere University, Tampere, Finland; 2 School of Engineering Science, Department of Separation Science, Lappeenranta-Lahti University of Technology LUT, Lappeenranta, Finland; 3 Faculty of Social and Health Care, LAB University of Applied Sciences, Lappeenranta, Finland; 4 Pedagogical Innovations and Culture, Tampere University of Technology, Tampere, Finland; 5 Finnish Defense Research Agency, Lakiala, Finland

**Keywords:** Holistically sustainable continence care, incontinence, sustainability transitions, single-use absorbent hygiene products (AHPs), incontinence pads, ecosystems

## Abstract

Incontinence is a common health issue that affects hundreds of millions of people across the world. The solution is often to manage the condition with different kinds of single-use continence technologies, such as incontinence pads and other absorbent hygiene products (AHPs). Throughout their life cycle, these fossil-based products form a remarkable yet inadequately addressed ecological burden in society, contributing to global warming and other environmental degradation. The products are a necessity for their users’ wellbeing. When looking for sustainability transitions in this field, focus on individual consumer-choice is thus inadequate – and unfair to the users. The industry is already seeking to decrease its carbon footprint. Yet, to tackle the environmental impact of single-use continence products, also societies and health systems at large must start taking continence seriously. Arguing that continence-aware societies are more sustainable societies, we devise in this article a society-wide working definition for holistically sustainable continence care. Involving dimensions of social, ecological and economic sustainability, the concept draws attention to the wide range of technologies, infrastructures and care practices that emerge around populations’ continence needs. Holistically sustainable continence care is thus not only about AHPs. However, in this article, we examine holistically sustainable continence care through the case of AHPs. We review what is known about the environmental impact AHPs, discuss the impact of care practices on aggregate material usage, the future of biobased and degradable incontinence pads, as well as questions of waste management and circular economy. The case of AHPs shows how holistically sustainable continence care is a wider question than technological product development. In the end of the article, we envision an ecosystem where technologies, infrastructures and practices of holistically sustainable continence care can flourish, beyond the focus on singular technologies.

## Graphical abstract

**Graphical abstract:**
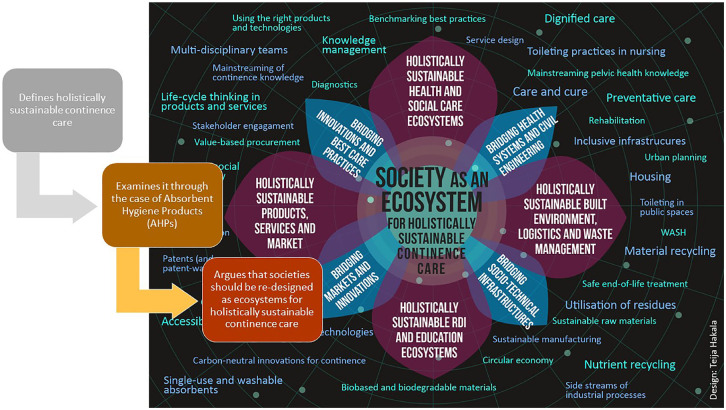

## Introduction

Incontinence, that is, an involuntary leakage of urine or faeces, is a common health issue which affects hundreds of millions of people worldwide. It has been estimated that 10.0% of all women and 37.1% of those over 50 live with urinary incontinence (UI).^1,2^ Respectively, the prevalence of UI among community-dwelling men has been estimated between 4.8% to 32.2%, depending on age^1^. Faecal incontinence (FI), in turn, affects 8.1%–8.9% of community-dwelling men and women,^3^ being also one of the most common reasons for nursing home admission.^1^ Many types of incontinence can be treated, mitigated, and even cured, and different engineering innovations have helped to develop treatments over the past decades. These include, for example, biofeedback and other diagnostic and rehabilitative technologies, innovations to support selfcare, sacral and tibial neuromodulation technologies, as well as mini-invasive and more invasive surgical technologies (see also Culmer et al.^4^). However, not everyone has access to adequate care, cure, and technology. While the stigma attached to the condition hinders people from seeking medical help,^5–7^ in societies at large, there is also a lack of knowledge about different forms of incontinence and their causes, including in primary health and social care services.^8,9^

Even though continence is a daily concern for every human being from the moment of birth to death, the training of health professionals does not customarily include holistic training in continence issues. Consequently, treatable conditions are not always properly screened, assessed, diagnosed and treated.^9,10^ When incontinence cannot be cured – or the person has no access to adequate assessment and treatment options – the standard solution is to manage the condition with continence products. Various continence management products exist,^11^ yet the most common is the single-use body worn absorbent incontinence product commonly known as the incontinence pad.^12^

Each day, humanity uses and disposes of hundreds of millions of these fossil-based, single-use absorbent hygiene products (AHPs). This (as well as other single-use continence technologies) increases the environmental burden of social and health care in multiple ways, adding to the extraction of raw materials, increasing water usage, pollution and greenhouse emissions, with damaging effects on planetary health.^13–15^ The ecological burden of continence products is difficult to discuss, as it may feel like putting the blame on environmental degradation on people who live with incontinence and need the products. At the outset, thus, we want to emphasise that this is not our aim. The environmental burden of continence technologies is *not a matter of individual consumer choice*. People living with incontinence depend on continence products for their daily wellbeing and hygiene, and they should not feel guilt for using AHPs or any other continence technologies, such as catheters, faecal devices, sheaths or stoma bags. Holistically sustainable continence care and engineering are *societal challenges*. Societies, at large, need to take continence seriously and, in collaboration with industry, build the kinds of ecosystems where continence needs are met with good care, and where a good and ecologically sustainable life with incontinence is possible. Accordingly, we need concepts and visions on which holistically sustainable and continence-aware societies can be built. This is what we seek to provide in this article.

While it is important to consider also other continence technologies, the impact of AHPs alone on the environment is significant, which means that sustainable approaches to continence care are urgently needed. The overall waste produced from AHPs is difficult to estimate, as knowledge and official statistics remain scattered.^13^ However, it is known that billions of absorbent hygiene products (AHPs) are disposed of each year.^16^ While this estimate also includes menstrual products and infant nappies, the amassing of AHP waste is increasing particularly in the incontinence product sector. Between 2010 and 2021, sales of adult incontinence pads increased 57%, accounting for around 38.97 billion units across the global market in 2021. Over the next 5 years, the adult incontinence pad market is expected to grow at compound annual growth rate (CAGR) of 2.5%.^17^ In a recent review on the waste-management of AHPs in different parts of the world, Velasco Perez et al. estimated that absorbent incontinence products form an average of 4.8% of municipal waste in OECD countries (by weight). In comparison, in a lower income setting of Latin America where the population is younger and access to incontinence products likely to be lower, 3.3% of waste came from absorbent incontinence products. In countries with rapidly ageing demographics, these estimates were considerably higher than the average. For instance, in Japan, it was estimated that 10.1% of municipal waste comes from adult incontinence pads,^13^ and in a study conducted in England, Takaya et al. showed that over 70% of healthcare institutions’ ‘offensive waste’ (i.e. ‘non-hazardous waste that contains body fluids from non-infectious humans’) is comprised of AHPs.^18^

In discussions of continence care and technology, sustainability is often approached from specific disciplinary definitions, with limited focus on, for example, biodegradable or reusable products, sustainable waste management, or better care pathways. In this article we bring together knowledge from several disciplinary standpoints, in order to pave the way towards continence-aware societies which, we suggest, could lead to ‘radical historical change in the form of a “Deep Transition”’.^19^ Our article has three aims. First, in section 2, *we provide a working definition for holistically sustainable continence care* that comprises elements of social, ecological and economic sustainability and goes beyond the development of individual technologies and care practices. This is a call for further research and discussion and, as such, still an abstract definition. In the scope of one article, it is not possible to discuss, what our model of holistically sustainable continence care would mean for all the technologies, infrastructures, and practices that are relevant for quality continence care for different patient populations in different contexts. Thus, our second aim is to *exemplify what holistically sustainable continence care would mean in the case of the most commonly used continence technology, namely, disposable AHPs*. The example of AHPs shows how the sustainable future of continence care is never a question of singular technological innovations, but about wider socio-technical ecosystems where sustainable infrastructures, technologies, and care practices must support one another. *The third aim of the article is to envision how an ecosystem for holistically sustainable continence care would look like*. This we do this in section 4.

As for research materials, our working definition on holistically sustainable continence care (section 2) is informed by a Finnish research project ‘Emergent un/sustainabilities of care: The global political economy of the adult incontinence pad’ (Pad Project).^20^ This is an extensive qualitative research project, with data consisting of diverse research materials coproduced with key stakeholders in Finland and internationally between 2019 and 2023, including, for example: key informant interviews (*n* = 36); innumerable informal email and personal exchanges; workshop data collected with health and medical professionals, policymakers, patient-advocacy groups, and the industry; utopia stories on ideal care pathways written by health professionals (*n* = 60); and autobiographical ‘pad stories’ stories written by persons who use adult incontinence pads themselves or help others to use it (*n* = 33). Providing a proper empirical analysis of such a variety of qualitative data goes beyond the scope of this article. We do not therefore cite these materials directly, even though the working definition builds on the data and previous analyses thereof.^21–23^ In our review on the present and future of AHPs (section 3), in turn, we rely on existing academic literature (especially review articles), market outlook reports, as well as on unpublished research of the authors Laatikainen, Tella and Koljonen on bio-based and biodegradable materials since 2017. Our argument on the wider ecosystems that holistically sustainable continence care requires (section 4) builds on all the authors’ collaborative thinking over the past 4 years.

## A working definition for holistically sustainable continence care

In 1987, the Brundtland report defined sustainable development as development that ‘meets the needs of the present without compromising the ability of future generations to meet their own needs’.^24^ Drawn from this definition, and bearing in mind the need to balance social, ecological and economic sustainability with ‘[t]he intimate intertwinement between ecological balance and human health’^25^ (p. 2), we define holistically sustainable continence care as follows:

- *Holistically sustainable continence care is socially, ecologically and economically sustainable. It meets the continence care needs of the present without compromising the ability to meet the needs of continence care in the future*.
- *It accounts for the continence needs of all people as they change over their individual lifespan.*
- *Comparing the environmental impacts of alternative care pathways beyond the choice of products, holistically sustainable continence care integrates life-cycle thinking not only in product development, use and disposal, but also in care practices.*
- *Holistically sustainable continence care requires a radical change and deep transition in health systems. Furthermore, the socio-technical infrastructures of societies at large must be built to meet the needs of continence care and technology.*
- *Because continence concerns all human beings at all times of life, questions of continence are to be integrated into the design of the built environment, logistics, sanitation and waste management.*
- *Holistically sustainable continence care emerges upon existing practices, technologies, and infrastructures, and therefore its specific meanings must be defined separately in each society. The prerequisite for doing so is putting continence at the centre of (re)designing sustainable societies.*
- *In holistically sustainable continence care, preventative care and better diagnoses, followed by appropriate treatment and rehabilitation, and the tailoring of continence products to individual needs saves money and the environment, while improving the quality of life for people with incontinence.*

**Figure 1.**
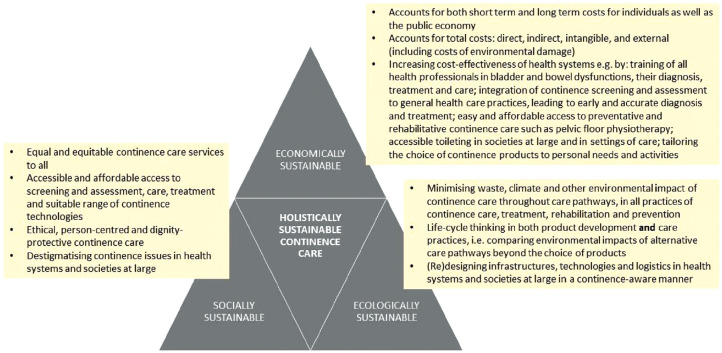
A model for holistically sustainable continence care, depicted as a simplified triangle-model for sustainability.

As depicted in Figure 1, our working definition for holistically sustainable continence care consists of three triangles of ecological, social and economic sustainability, each supporting one another in a way that it becomes possible for holistically sustainable continence care to emerge in the centre. Here, it is important to note that social and ecological sustainability, placed at the bottom of the diagram, form the basis of holistic sustainability. This means that the economic sustainability of continence care relies on social and ecological sustainability: If either of the bottom triangles were eroded (e.g. through inadequate and inequitable access to treatment and medical technologies, or by an unsustainable use of natural resources), the economic sustainability of the system would be shaken as well. Consequently, the equilibrium of holistically sustainable continence care would collapse, impacting upon the economic sustainability of the entire health system in multiple ways.

Furthermore, the size and balance of each of the smaller triangles is co-dependent on what happens in the premises of the other two triangles. At the bottom left-hand side, socially sustainable continence care relies on an equal and equitable distribution of continence care, preventative and rehabilitative services, treatments, and necessary technologies across the population. For socially sustainable continence care, these should not be privileges of a certain group such as rich or working age populations. Socially sustainable continence care is accessible, person- and relationship-centred, ethically delivered^26^ and dignity-protective for all,^27,28^ regardless of age, sex, gender, class, race, ethnicity or any other social determinants of health. Social sustainability, however, is co-dependent not only on the economic resources of the health system, but also on ecological sustainability. Namely, the wider socio-technical infrastructures, technologies and logistics of care delivery need to be ecologically sustainable throughout their life cycles, or else the care of some brings harm to others, including the planet (see sections 3 and 4 of the article). For instance, if the waste management infrastructures do not support the safe disposal of used continence products – be they single-use AHPs as discussed in this article, or any other continence technologies – the environmental damage caused exposes other people to health risks, which in turn has wider impacts on both the social and economic sustainability of health systems at large.

Both social and ecological sustainability arguably require sustainable continence economics.^29^ According to Hensher, particularly in high-income countries, the economic evaluation in health care focuses ‘overwhelmingly on Health Technology Assessments (HTA)’,^30^ (p. 2) where new products and devices are approved for public funding. However, HTAs tend not to include calculations on environmental impacts or subsequent negative externalities, where certain costs of the use of the technology (such as environmental damage) are externalised to other parties within or outside the health system in question.^30^ In holistically sustainable continence care, considering the environmental costs is key to the assessment of alternative care practices and continence technology options. Indeed, as health systems are urgently searching for ways to reduce their ecological footprint, engineering innovations for continence technology that help to minimise the negative externalities of environmental damage are likely to provide a market advantage.

Our definition for holistically sustainable continence care is arguably complex, raising perhaps more questions than it answers. Yet, understanding this entire complexity is a pre-requisite for sustainable care practices, and it also reveals possibilities. It is known, for example, that the economic costs of incontinence on public health systems are enormous. Already in 2008, the United States spent more on the treatment and diagnostics of incontinence than on coronary bypass surgeries and dialysis combined.^31^ In Canada, the annual costs of incontinence were estimated at 8.5 billion Canadian dollars in 2014.^32^ Investing in socially sustainable continence care by developing adequate and accessible preventative and rehabilitative services, accessible toileting (also when help is needed, as in ‘disability-associated incontinence’), early and accurate diagnostics, and developing technologies to support these areas could help reduce the economic costs of public health by mitigating or curing incontinence. Simultaneously, the need for AHPs and other single-use products would decrease – decreasing also ecological burden. The aims of social, economic, and ecological sustainability are thus not necessarily in tension with, but can also support one another.

It is not self-evident to depict holistically sustainable continence care as we do in this article. We could have, for instance, placed economic and social sustainability at the bottom, with the presumption that without adequate economic resources and social equality and equity, ecologically sustainable development is not possible. This alternative, however, would not have adequately emphasised the *factual limits of the planet* and human life,^33^ which tend to be forgotten when economic sustainability is prioritised. If the ecological and social sustainability of care collapse, the very basis on which economies rely is eroded. This includes, on one hand, the social infrastructures of care that sustain human life and, on the other, the health of the planet, without which human life will perish. On a dying planet, the ecological dimension of sustainability is the most urgent issue to attend to.^34^ This is why we now turn to the case of AHPs, the wiser production, use and disposal of which can significantly reduce the ecological impacts of incontinence across societies.

## The case of AHPs

### The impact of care practices on material usage

To our knowledge, there are no existing studies of holistically sustainable continence care that address the entire scope of the challenge. However, in different scholarly disciplines from health sciences to engineering, there are some studies on the environmental sustainability of continence technologies.^13–15,18,35,36^ Drawing on the ‘three R approach’ to sustainability (‘reduce, reuse, and recycle’), and including also other continence technologies such as intermittent catheters and toileting devices, Macaulay et al. have for instance asked whether sustainable continence products are a realistic option. They emphasise that ‘reducing or curing incontinence is the most desirable goal’ in continence care, and therefore ‘assessment interventions to prevent and reduce incontinence should […] be the highest priority’^35^ (pp. 32–33). This view is in line with our working definition for holistically sustainable continence care. Or, as Thompson Brewster et al. aptly put it, ‘a green engineering approach’ would be to develop health care policies and practices addressing adult incontinence, as that would reduce the amount of AHP waste ‘at its source’^15^ (p. 36) by means of better care and cure.

Another challenge is in mainstreaming knowledge on products and their environmental impact in health systems, both in institutional settings, such as nursing homes and hospitals, as well as in community care and practices of self-care. Namely, in cases where incontinence cannot be cured,^37^ the reduction of pad waste begins with tailoring the choice of the products to individual needs. With the present technologies in both products and waste management, this is the most effective way of reducing the environmental impact of AHPs,^14^ and the same applies most likely also to other continence technologies. Macaulay et al. also note that sometimes ‘[f]ewer highly absorbent pads of the most appropriate design may be more effective than several less-absorbent or less-effective ones’^35^ (p. 33). Sometimes this is true, yet a more absorbent product is not automatically more sustainable. Tailoring the number, quality, and type of pads per day is a complex problem that requires a highly personalised ‘mix-and-match’ approach,^35^ where the person’s daily activities and voiding patterns are matched with the right size, material absorbance and fit – and potentially also other available continence technologies – while including also care practices that support continence, such as adequate assistance in toileting where needed.^38,39^

Unfortunately, in present continence care practices, particularly in institutionalised settings, continence products tend not to be tailored to the users’ needs. In nursing homes and hospitals, it is not exceptional that products are purchased in bulk for all the clients, with the perception that one size and absorbance fits all, regardless of the person’s size, gender, weight, anatomy, or personalised patterns of voiding. If the product is the wrong size or type, this can lead to skin problems, leakages, soiled beds, consequently increasing environmental and financial costs of washing, as well as a poor quality of care. Sometimes the most absorbent products are chosen just in case to avoid leakages, particularly at the night-time. At other times, double pads are used for the same reason.

Such systematic practices of using too absorbent and the wrong size of products is highly unsustainable economically and ecologically, and often detrimental to the wellbeing of those who need the products. In the UK, Featherstone et al. have also shown how present ageist ‘cultures of care’ in hospital settings not only treat pads as non-personalised bulk material, but continent older persons are systematically made to use pads instead of helping them to go to toilet. This increases hospital-acquited incontinence post-admission, consequently increasing the aggregate pad use among community-dwelling older adults.^38^ Breaching the person’s right to dignity,^27^ this is socially unsustainable, but also environmentally and economically untenable.

As for nursing homes, in a recent life-cycle assessment (LCA) of body-worn absorbent continence products, Willskytt and Tillman examined the environmental impacts of more effective use of products through their customisation to the users’ needs. They found that the nursing home participating in the study would use up to two levels higher absorbance than would have been necessary, and that the large body-size (and not e.g. voiding patterns) of the client was associated with the need for more absorbent pads.^14^ After a measurement to customise the use of products for personal needs, the level of absorbance could be reduced for most clients. For some, also the size of the products could be reduced. As smaller size and less absorbent products require less raw materials and produce less waste in both production and at the end of life, the environmental impacts of the intervention were remarkable, resulting ‘in a 23% decrease in global warming potential, a 20% decrease in fossil depletion and a 18% decrease in land use’^14^ (p. 20). Namely, the study found that, in all the products and practices examined, production processes dominated the environmental impact, with the material production process making up 60%–90% of the ecological footprint. Thus, all care practices and product developments that reduce material use can significantly improve the environmental sustainability of continence care.^14^ Because the incontinence pad is such a mundane, frequently and widely used product, even small changes in the choice of products can quickly scale up on the level of health systems, thus helping to reduce the systems’ overall environmental footprint. However, in attempts to reduce the material consumption, the dimension of social sustainability must be borne in mind, as the reduction cannot happen by means of barring persons living with incontinence from access to the technologies they need.

Willskytt and Tillman also found potential in products where part of the product could be reused (a reusable pant with a single-use absorbent insert), particularly in terms of global warming and fossil depletion, although the impact on land-use compared to the disposable products remained negligible.^14^ In the reusable and hybrid systems, the outer layer is washable or reusable, whereas the inner layer is single-use. The outer reusable layer can be made of cotton, but washable and reusable absorbent pads can also be made entirely from bio-based viscose.^40^ As noted by Macaulay et al., washable and reusable continence technologies can be an alternative for single-use products for some users, particularly when it is possible to ‘mix and match’ the choice of products to the user’s personal needs and activities.^35^ Indeed, it is also important to note that washable, textile-based absorbent incontinence products never completely vanished from the markets after the development of single-used products. Various combinations of pads and topped underwear consisting of water-permeable top sheets, urine absorbing textiles and waterproof backing have been designed and developed to meet even substantial urine losses of both women and men.^41^ These days, many of the companies that produce period underwear, as well as some brands otherwise known for their single-use AHPs, sell washable incontinence briefs for lighter urine leakages. In addition, there are washable inserts available for heavier leakages, too.^42^ The companies that sell washable products also provide solutions for carrying the used products and advice for washing the products, which have earlier been regarded as the main challenges for the appeal of the washable products.^41^

It needs to be noted, however, that in institutional settings of care, reusable and washable products are unlikely to provide a sustainable alternative. This is not only due to the already existing staffing shortages in these settings, but also due to the health risks involved in processing large amounts of textiles soiled with human secretions that may include medical residues, resistant bacteria, and viral loads.^18^ To our knowledge, the relationship between the appropriate use of AHPs and the emergent threat of antimicrobial resistance (AMR) to health systems has not been studied. In engineering for holistically sustainable continence care, AMR is an issue to consider, particularly when developing solutions for institutional settings.^43^

An emergent technology in absorbent products is the use of sensors and smart solutions as an indicator for wetness or odour, or in digital continence assessments, such as tracking voiding patterns in real-time.^4,44^ The growth in this sector is fast, and the demand for smart pads is projected to grow at a CAGR of 19% in the years to come.^17^ These products can help increase patient privacy and comfort, decreasing the need for the caregivers to check manually whether a pad needs to be changed. They may also serve the aims of ecological and economic sustainability, as the sensor can aid sustainable material usage by allowing pad change at the optimal time, and the digital continence assessment technologies can help tailor the choice of products for individual needs. In this regard, smart pads may well help in the advancement of holistically sustainable continence care.

However, these products may also have potential negative environmental impacts, which ought to be further investigated, by means of LCA for example. First, most sensor technologies require metals or other non-renewable natural resources retrieved through mining. Therefore, adding sensors to the pad means adding new non-sustainable elements into the product’s life cycle, particularly if (and when) the materials cannot be recycled. Yet, nothing prevents designing the sensors in pads to be as reusable as possible. Secondly, while being an increasing trend across health systems, the further digitalisation of health care products may increase energy consumption, and thereby the ecological footprint of the system. As noted by Lokmic-Tomkins et al., ‘[w]hile global healthcare is the fifth largest polluter on the planet, accounting for approximately 4.4% of global greenhouse gas net emissions, it is less well known that information and communication technology emissions account for 3.7% of global carbon emissions’^45^ (p. 2136). The same authors show that the ecological footprint of digitalisation is not systematically assessed when introducing new smart health technologies to health systems. Thus, to design smart pads sustainably and to determine their role in ecosystems of holistically sustainable continence care, further research on their overall ecological footprint is required.

### The promise of biobased and biodegradable products

Incontinence pads are made of fossil-based plastics or nonwovens, forest-based cellulose fluff, and fossil-based superabsorbent particles (SAPs), consisting of 6–10 layers of different nonwoven material. The inside and outside layers have been typically made of polypropylene and or polyethylene, whereas the acquisition layer under the top layer has been made of polyester or polyethylene. The thickest middle-layer forms the core of the product, consisting of cellulose fluff which forms about 43%–59% of the product, and super adsorbent polymers (SAP) which make up about 14%–27% of the product.^18^ A major challenge to the environmental sustainability of AHPs comes from the SAPs, which are water-adsorbing polymers also known as hydrogels.^46^ They are capable of absorbing and retaining extremely large amounts of liquids relative to their own mass.^47^ For example, the sodium polyacrylate containing diapers can absorb water approximately 200–300 times it weight.^48^ The SAPs used in continence products absorb up to 300 times their weight in aqueous fluids,^49^ although – as corrected by one of the anonymous reviewers of this article – the industry typically considers the retention capacity of SAP (under load), which is rather in the range 30–50 times.

Helping to make the product lighter and more comfortable to wear for the users, SAPs are an important ingredient in single-use AHPs. The SAPs used in incontinence pads are usually based on acrylic acid-acrylamide polymer mixtures which are neither biobased nor biodegradable – that is, they are fossil-based, non-renewable raw materials. Compared with the other materials used in AHPs, SAPs are the most harmful in terms of their contribution to global warming and fossil depletion.^14^ Chen et al., for instance, note that plastics cause a major contamination problem in the biosphere of the planet, with over 80% of the world’s plastic accumulated in landfills or released to the environment. This is particularly the case with microplastics such as SAPs, as they can absorb organic pollutants that transfer along the food chain, with damaging impacts on human and non-human life. Taking a 95% global share of the rapidly growing SAP markets, single-use AHPs are the biggest application area for these hydrogels.^47^ The AHP industry and related fields of engineering thus have a big role to play in developing more sustainable alternatives, and thereby decreasing the overall release of SAP-originating microplastics in the environment.

The global AHP industry has already taken on this challenge. Research on biobased and biodegradable materials for AHP is developing fast to replace the fossil-based nonwovens and SAP.^50^ Here, the term ‘biobased’ refers to renewable and non-fossil materials deriving for instance from plants or forest industry side-streams. Turning to biobased raw materials in AHP production increases environmental sustainability by reducing fossil depletion. However, not all biobased SAPs are biodegradable, whereas some fossil-based SAPs can be.^47^ Commonly, biodegradability is a benefit only if waste can be sorted and composted. In sustainable product development, these distinctions are to be borne in mind, along with the questions of waste-management technologies (to which we return in the next section).

To decrease fossil depletion, biobased SAPs are constantly being developed, for example, from chitosan, starch, carrageenan, or cellulose. Starch-based SAPs can be corn starch-based, AzuraGel™, or wheat gluten proteins derived from wheat starch.^51^ In addition, TEMPO oxidised cellulose nanofibers^52^ and recycled cellulose, where waste cardboards have been bleached using peroxide, carboxymethylated and crosslinked with citric acid, can act as SAP material.^53^ However, we do not know to what extent these alternative SAPs have been tested in the adult incontinence product industry. Although the cellulose-based superabsorbents in personal care applications have been widely studied in recent years, there are still challenges that hinder their wider commercialisation, for instance in the management of materials, performance of hydrogels, and in the production costs.^48^ In baby diapers, synthetic polymers have been combined with bio-based polymers and applied with the purpose to improve the sustainability of the synthetic SAPs.^54^

Simultaneously, the industry is looking for biobased alternatives to AHP materials other than SAP. Biodegradable fibres such as cotton, rayon, hemp, bamboo viscose and biobased plastics have been applied in menstrual products, and in some children’s products, but less so in incontinence pads.^55^ However, for light incontinence, pads and liners made with bamboo fibre and corn-based polylactic acid (PLA) were launched by Jude earlier in 2022. PLA is a biobased and biodegradable plastic refined from renewable and biobased raw materials.

A central challenge for developing biobased and biodegradable products for heavier incontinence is that the new products should meet the same level of absorbance and be as comfortable to use as the products with fossil-based SAP. Furthermore, the manufacturing cost of biobased plastics is 20%–100% higher than traditional fossil-based plastics,^56,57^ which hinders innovation and market entry. The manufacturing processes of natural fibres are also cost and energy intensive, which upsurges the price of the final products.^58^ Converting old production lines suitable to the new, more sustainable innovations may also be challenging, which hinders the scaling up of production where new products could otherwise be made available. Similarly, existing standards may sometimes slow down the development of more sustainable products. This happens, for instance, when the new technology has performance features, which the existing standards cannot detect.^59^ Nevertheless, new standards are being developed, and the AHP industry also seeks to increase sustainability by other means, for instance by using renewable energy sources, minimising the production of waste in the production process, and developing the recycling processes for manufacturing waste.^14^

### Waste management of AHP: Present practices

As the previous section shows, the development of biobased products may soon be possible in single-use absorbent incontinence products. This is important, as replacing fossil-based materials is pivotal for holistically sustainable continence care. However, the environmental impact of AHPs depends not only on the raw material usage in the production phase. At the end of life of the product, what matters are the waste management infrastructures, logistics and technologies that are available in the institutional and societal context where the products are disposed. Velasco Perez et al. have conducted a global review of AHP waste management options. Presently, the options for processing AHP waste are incineration (waste to energy), anaerobic co-digestion of AHP with other biodegradable materials (waste to biogas and digestate), mechanical-biological treatment, landfill, or illegal dumpsites. The last mentioned is the most harmful option, yet common particularly in low-income countries.^13^

With present technologies, incinerating AHP waste to energy is one of the more environmentally sustainable alternatives. Its relative sustainability, however, depends on the technologies used in the incineration plants, for instance their energy systems and emissions, and whether the emitted carbon can be captured and reused.^60^ In the EU and Japan, incineration of AHP with other non-recyclable municipal waste is the most common practice. When landfilling is not an option due to legal restrictions (as in the EU), incineration is the cheapest option, and it also has the benefit of killing the pathogens in the waste. In Toronto, Canada, there are also facilities which produce biogas through a process where the AHP waste mixed with biodegradable materials is digested in anaerobic conditions. This, too, is one of the more environmentally sustainable alternatives.^13^

Landfilling AHP waste is environmentally harmful, yet still a prevalent practice in many industrialised societies such as Australia. In a recent study by Thompson Brewster et al. , it was estimated that the methane emissions from AHP waste can account for up to 10% of the total Australian landfill emissions in 2020–21.^15^ With methane being an 80 times more powerful greenhouse gas in the short-term than carbon dioxide, this adds considerably to climate change.^61^ Thompson Brewster et al. further emphasise that, if the waste is landfilled instead of sending it to alternative waste management facilities, biodegradable products are not likely to reduce the negative environmental impact. In fact, they could potentially even *increase* the greenhouse gas and leachate impacts.^15^

To understand how biodegradable AHP could be environmentally harmful when landfilled, it is important to understand the concepts of biodegradability and composting.^62^ Affected by microbes and enzymes, all biodegradable plastics (both biobased and fossil-based) degrade through a biological process into carbon dioxide or methane, water, and biomass.^47^ Composting, in turn, is the biodegradability of material in industrial or home compost at a certain temperature and humidity. This process is different from the above-mentioned anaerobic digestion, where organic waste is turned into biogas and digestate. The effect of composting can be observed as the degree of degrading or the carbon dioxide produced, and there are regulations and standards that define biodegradability. For instance, the compostability of plastic has been determined in standard EN 13432 according to which material should be degraded at 58°C over 3–6 months so that 90% of the carbon in the material has turned into carbon dioxide, and 90% of the material should degrade under 2 mm within 12 weeks.^62^ Furthermore, the material should be harmless to the composting process and to the quality of the organic end-product, staying below a certain threshold for heavy metals. Overall, for an incontinence pad to compost (i.e. break down into organic material), there are several factors at play, including the composition of the product, the amount of oxygen, the presence of micro-organisms and soil quality. Landfill conditions are not optimal for the process, and even if they were, the process still produces significant greenhouse emissions and leachate impact.

Thus, for biodegradable products to be environmentally sustainable, they require a sustainable waste management infrastructure at the end of life. Industrial composting would require a segregation of the waste, and logistical solutions for waste collection and delivery to the waste management facility in case composting at home is not an option. Here, it is important that all materials are compostable. Typically, a product designed to be fully compostable cannot be recycled and vice versa.^15^ Indeed, when a compostable or biodegradable plastic product is composted, new plastic cannot be produced from it and the energy used to produce it is lost.^63^ However, recycling options of biodegradable plastics have been less studied.^64^ In the children’s diaper sector, Dyper together with partnering waste management sites provides a service for some cities in the USA, where disposed compostable diapers are collected from home and taken to an industrial composting facility. Here, the diapers are composted into soil used for non-agricultural purposes, or turned into soil amendment material by means of anaerobic digestion.^65^ Another example of the recycling of children’s diapers is the concept invented by the DiaperRecycle which won the RISE Innovation award in 2022.^66,67^ In the concept, the used diapers are collected from homes and further processed using thermal pressure hydrolysis, making it possible to recycle single-use AHPs. In DiaperRecycle, the plastics and fibre are separated and recycled. The super absorbent fibre is used to produce flushable cat litter.^66,67^

In the future of holistically sustainable continence care, societies may well have similar small-scale and large-scale systems in place for the composting, recycling, and revalorisation of used adult incontinence pads. However, there may still be some environmental risks to consider, which require more research. For instance, in relation to large-scale composting post-consumer AHPs, Takaya et al. argue that there is no adequate knowledge yet available on the impact of slowly biodegradable SAP on soils.^18^ Yet, there is future potential in recycling and revalorisation of AHP, to which we turn next.

### Recycling of AHPs and the possibilities of circular economy

The single-use absorbent incontinence products are difficult to recycle, due to the materials that are used.^35^ As the above examples of Dyper and DiaperRecycle show, several technological advances have been made, however, and new recycling technologies for AHPs enable the retrieval of valuable materials such as cellulose and mixed plastic from waste.^16^ This enables their use for other purposes, such as in the construction industry. Two of the leading AHP manufacturers in Europe (Essity and Procter & Gamble) have invested in research that explores recycling post-consumer AHPs into commercial chemicals and fuels.^18^

Velasco Perez et al. discuss different examples for small-scale initiatives for AHP recycling. In the UK, there has been a commercial initiative, where a company called Knowaste offered waste treatment services for social and health care providers. Also, in Northern Italy, a plant serving 50 municipalities can recover 150 kg cellulose, 75 kg of mixed plastic, and 75 kg of SAP per metric tonne of processed AHP waste. The processes involve a sterilisation of the waste, shredding, washing, a chemical treatment to deactivate the SAP, and the separation of components which are then sold for recycling.^13^ Such SAP recovery technologies could be of value in the future, given that its initial production is both energy and resource-intensive.^18^

Velasco Perez et al. identify several technologies that have been tested in laboratory conditions, but which would need further research for large-scale application. These include, for example, (1) small in situ facilities that would degrade AHP within days; (2) degradation of AHP with an edible fungus, producing forage for agriculture as the result of the process; (3) production of biohydrogen by dark fermentation; (4) production of combustible pellets to be used in a biomass boiler; (5) production of pyrolysis gas and coat pellets; and (6) the valorisation of AHP waste as a viscosity modifying agent for cement grouts and concrete.^13^

The research on AHP waste recycling suggests that for scaling up the new innovations, several conditions must be met. For example: (1) the recycled materials gained from the process ought to be targeted towards non-food related purposes such as the construction; (2) there has to be a sufficiently steady demand for the recycled materials retrieved, as well as a constant and sufficient provision of material to the recycling facilities; (3) government incentives and initiatives are needed to develop waste management systems and logistics to serve the recycling of AHP, including the separate collection and storage of AHP waste; (4) landfilling of AHP should be banned or made inhibitively expensive by fees, and (5) the costs of the recycling process should be competitive compared to incineration.^13,15,18^ Many of these conditions point to the role of states and municipalities to create legislation and policies that enable and steer a more sustainable process of AHP waste management.

Successful examples of policy drivers and incentives exist. Thompson Brewster et al. , for instance, identify the lack of legislative restrictions in Australia as a disincentive of finding alternatives to landfilling.^15^ They contrast this to the European context, where the European Union Landfill Directive^68^ limits the disposal of biodegradable materials to landfill, which includes AHP. The Directive has been a key driver for the EU member states to develop alternative waste management facilities, cutting the amount of landfilled municipal waste in the EU by more than 50% by 2020.^69^ In Denmark, Germany, Finland, Belgium and Sweden, landfilling of municipal waste had virtually ceased by 2020. In some countries, the change has been significant, and for instance in Finland, landfilling is close to 0% today, whereas 50% of municipal waste was still landfilled in 2010.^70^ In addition to national or supranational legislative measures, local governments can also utilise various policy tools as drivers for change, such as ‘extended producer responsibility systems, bans, levies, ecolabelling, or a combination of these’ as means to reduce the burden of AHP waste.^13^ (p. 767)

Another European level example potentially of relevance to holistically sustainable continence care is the Directive 2019/904 to act against plastic pollution in oceans and seas, also known as the single-use plastic (SUP) Directive.^71^ It bans certain single-use plastic products such as plates, cutlery, straws, cups food and beverage containers, and oxo-degradable plastics which constitute about 70% of all marine litter items, and can no longer be placed in the market. It is possible that some single-use AHPs will also be included under the ban in the future, and the Directive is due to be assessed by the Commission by July 2027. In the interim, the Commission is preparing to propose EU-wide rules and restrictions on packaging to tackle the constantly growing source of waste.^72^ This too will have an impact on the AHP industry. As an example of bans in other countries, Vanuatu has prohibited the use of single-use baby diapers, although the ban has been placed on hold since the double crisis of COVID-19 and cyclone Harold.^73^

## Envisioning ecosystems for holistically sustainable continence care

Our model of holistically sustainable continence care requires the rethinking of policies, built environments, socio-technological infrastructures – in fact, everything in society – in ways that serve continence needs. This should not be difficult. After all, continence is the one thing that concerns all people, every day, from birth to death, kings and paupers alike. Yet, the sustainability transition required is no less radical than building infrastructures for a modern sanitary sewage system that were once needed in industrialising societies. Although even the British Association of Urological Surgeons’ history website sees ‘going to the toilet, whenever and however you do it’ as something that is ‘common to all of us’, with ‘flush toilets […] here to stay’,^74^ this is categorically untrue. Hundreds of millions of people need technologies other than the flush toilet to manage their continence. The time has come to develop societies sustainably in ways that recognise all kinds of continence needs. We argue that this would lead not only to better continence care, but also to more sustainable health systems and societies overall.

**Figure 2.**
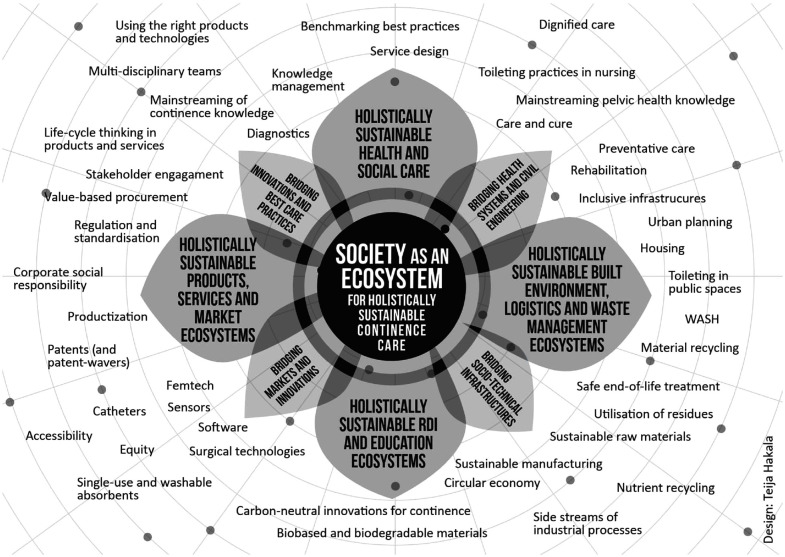
Society as an ecosystem for holistically sustainable continence care (Graphic Design: Teija Hakala).

But how do we get there? As the case of AHPs shows, the role of legislative measures and other policy drivers is pivotal in catalysing change. Echoing the ‘incontinence thematic innovation ecosystem’ that the World Federation of Incontinence and Pelvic Problems launched as part of their 2022 World Continence Week programme,^75^ we argue that policies must strive to develop society-wide ecosystems for holistically sustainable continence care. As implied in Figure 2, these ecosystems would encompass the entire society, being wider in scope than systems of health and welfare, or the continence technology industry. Yet, in each corner there are needs for engineering innovations.

What do we mean by ecosystems for holistically sustainable continence care? Initially, the term ecosystem has been used to describe biological communities consisting of all the different living organisms (including humans) in a certain area. These organisms are interconnected, interacting with non-living components like air, water, and soil. It is typical that the boundaries of different ecosystems cannot necessarily be recognised, as ecosystems overlap, interact and merge with one another^76^ (pp. 591–592). In healthcare, ecosystem thinking highlights that health and wellbeing related needs and problems cannot be solved in isolation. Similarly in entrepreneurial ecosystems – including in the field of continence technology – the needs of different industries serve and co-constitute one another. Furthermore, health care, as well as entrepreneurial and industrial systems are all fully contained within societal systems, which in turn are completely contained within the broader ecological system, where nature and the environment are the most fundamental to life. For this basis of life to be preserved in the current context, we propose *that societies should be redesigned into an ecosystem for holistically sustainable continence care*.

As depicted in Figure 2, consisting of various smaller and overlapping ecosystems, society as an ecosystem of holistically sustainable continence care would be a multidimensional and multisectoral ecosystem of ecosystems including:

1. holistically sustainable health and social care ecosystems;
2. holistically sustainable ecosystems for built environment, logistics, and waste management;
3. holistically sustainable research, development, innovation (RDI), and education ecosystems;
4. holistically sustainable product, services, and market ecosystems.

These four sectors of distinct, yet multiply connected, ecosystems would all be continence-aware, and hence serve the varying needs of social, ecological, and economic sustainability in continence care that have been described in the first section of this article. To be holistically sustainable, these four ecosystems are partially entangled with and co-dependent on one another. They share the same overlapping actor-networks and appreciate each other’s roles and practices, since the sustainability of each sector depends on the sustainability of the others. What connects them is their joint purpose of helping societies meet the needs of continence in a sustainable way.

This utopian multisectoral ecosystem can be depicted as a flower that flourishes in a multidimensional space (Figure 2). Its four bigger petals represent the four ecosystems or sectors mentioned above. These are connected by four smaller petals that serve as interfaces between their tasks and practices, ensuring shared learning, communication, the co-creation of knowledge, as well as knowledge management across the ecosystem boundaries. In the surrounding space around the flower, the shared actor-network, different stakeholders, processes, and materials are in constant motion, connecting and reconnecting with one another to serve the varying needs of the society as a holistically sustainable continence care ecosystem, and its sub-systems. In Figure 2, the flower is depicted from above, so its stem is hidden from view. However, the flower – that is, society as an ecosystem for holistically sustainable continence care – is deeply rooted in the soil of our planet, which must be preserved for any of the other elements to stay alive.

## Conclusions

On average, health care produces 5.5% of the total greenhouse emissions in OECD countries, China and India, and up to 9.8% in the USA.^30^ The scholarship on sustainable health care has addressed this impending challenge for some time.^77,78^ Yet, despite the significant environmental impact of single-use continence technologies, such as AHPs, continence care has not been comprehensively addressed in this scholarship. In this article, we have provided an over-arching working definition for holistically sustainable continence care, addressing technologies, infrastructures and practices of care and comprising the dimensions of social, ecological, and economic sustainability. We have then examined the case of AHPs through the lens of holistically sustainable continence care, addressing the impact of care practices on material consumption, the promise of biobased and biodegradable products, waste management infrastructures, circular economy, and the policy drivers required for sustainability transitions. We have identified strategies to reduce the aggregate material need for these products via better, person-centred care, where the choice of products is tailored to the user’s needs. We have also emphasised that the most sustainable way to reduce the environmental impact of incontinence is to prevent and treat incontinence and support continence, where possible, thus reducing the need for AHPs and other continence technologies.

Taken together, we have shown that for societies to get to holistically sustainable continence care, radical changes are required not only in health systems, but in all sectors of society. We argue that societies need to be transformed into ecosystems of holistically sustainable continence care, and we have envisioned how such constellation of ecosystems would look like. Imagining society as an ecosystem for holistically sustainable continence care may be utopian, but many scientific innovations begin with and are inspired by utopias. Redesigning societies around continence needs would lead to deep and radical sustainability transitions. With appropriate policy drivers and stakeholder collaboration, this change is possible. The building of ecosystems for holistically sustainable continence care depends not only on businesses or individual stakeholders, such as health professionals and health system managers, but also on local and national governments, on supranational actors such as the European Union, as well as the various intergovernmental organisations that drive the agenda of sustainable development. On all these policy levels, it is time to put the question of continence high on the agenda as a prerequisite for sustainable health systems and societies.

As noted by Culmer et al. , the challenge of continence technology is ‘not limited to the development of new technology, but extends to exploring ways to improve existing systems’^4^ (p. 2). Indeed, the complex system of holistically sustainable continence care provides opportunities for an entirely ‘novel engineering science’ to emerge,^4^ namely *holistically sustainable engineering for continence*. While it remains important to develop technologies to improve personalised care pathways, diagnosis, product usage and technologies of conservative and surgical treatment,^4^ our model of holistically sustainable continence care invites engineers in medicine to explore innovative linkages with other engineering disciplines, in relation to areas such as waste management, logistics, and the built environment. To get to holistically sustainable continence care, there is a need for radically transdisciplinary collaboration, while bearing in mind the dimensions of social, ecological and economic sustainability – and thereby the need of other scholarly fields, including social sciences and humanities.

Indeed, our discussion on the present and future of AHPs is but one example of the kind of questions that emerge, when continence technologies are assessed from a more holistic perspective. For ecosystems of holistically sustainable continence care to emerge, similar analyses of ecological sustainability should be done also to other continence technologies: accounting for their entire life-cycles, and how the life-cycles shape and are shaped by care practices on the one hand, and wider socio-technical infrastructures, such as waste management, on the other. In other fields related to medical engineering, further questions of holistically sustainable continence care emerge as relevant. Nursing and medical sciences must attend to questions of holistically sustainable continence care practices. Continence economics must ask, which *combination* of treatments, practices, and technologies lead to ecologically and socially sustainable spending. For the continence technology industry, the puzzle is about ecologically and socially sustainable business, and growth – or degrowth – that stays within the planetary boundaries,^33^ while seeking to meet the needs of those who live with incontinence. The challenge of holistically sustainable continence care cuts through the entire society – and this is why it would lead to deep and radical sustainability transitions across societies.
